# Altered sagittal plane mechanics is associated with Functional Movement Screen deep squat score

**DOI:** 10.4102/sajp.v79i1.1865

**Published:** 2023-07-27

**Authors:** Candice Macmillan, Benita Olivier, Natalie Benjamin-Damons, Wendy-Ann Wood, Oluchukwu L. Obiora

**Affiliations:** 1 Wits Cricket Research Hub for Science, Medicine and Rehabilitation, Department of Physiotherapy, Faculty of Health Sciences, University of the Witwatersrand, Johannesburg, South Africa; 2 Sports Exercise Medicine and Lifestyle Institute (SEMLI), Faculty of Health Sciences, University of Pretoria, Pretoria, South Africa; 3 Department of Physiotherapy, Faculty of Health Sciences, University of the Witwatersrand, Johannesburg, South Africa

**Keywords:** Functional Movement Screen (FMS), deep squat (DS), kinematic motion analyses, cricket, validity

## Abstract

**Background:**

The Functional Movement Screen (FMS) assesses the quality of movements, including the deep squat (DS), which is used in sports settings. The validity of the individual item scores has yet to be established.

**Objectives:**

To investigate the validity of the FMS DS by comparing the sagittal plane kinematics of participants who achieve different observer scores.

**Method:**

Seventeen injury-free, adolescent male cricket bowlers were assessed. The movement was captured using the Optitrack^®^ motion capture system. Simultaneously, observers scored participants’ execution of the DS according to the standard FMS scoring criteria. Participants were grouped into Group 1 (lowest score), Group 2 (altered movement mechanics) or Group 3 (perfect score) according to observer scores. Specific joint angles of each group were compared using the Kruskal–Wallis and Mann–Whitney U tests.

**Results:**

There were significant differences in the degree to which the femur passed the horizontal between Group 3 and Group 1 (*p* = 0.04, *r* = 0.61) and Group 2 and Group 1 (*p* = 0.03, *r* = 0.66) and the difference in the degree to which the torso was kept vertical between Group 3 and Group 1 (*p* = 0.02, *r* = 0.66) and Group 2 and Group 1 (*p* = 0.02; *r* = 0.72).

**Conclusion:**

Kinematic differences exist between participants who achieve different observer scores for the FMS DS.

**Clinical implications:**

While differences in sagittal plane kinematics have been observed in participants scoring high on the FMS DS and participants scoring low, further investigation into the validity of the frontal plane kinematics is warranted, as well as the concurrent validity of the individual scoring criteria.

## Introduction

The Function Movement Screen (FMS) is a pre-participation screening instrument assessing seven fundamental movements, including the deep squat (DS). It is designed to identify limitations, asymmetries and compensatory movement patterns (Cook, Burton & Hoogenboom 2006). Its application is based on the construct that a solid base of good quality fundamental movement will protect the individual from injury. Each test is scored as ‘3’, ‘2’ or ‘1’ according to specific criteria, with ‘3’ being a perfect score and ‘1’ being the poorest performance. A score of zero is applied if the individual experiences pain at any time during the test (Cook et al. 2014). To meet the prescribed criteria and attain a score of ‘3’, an individual requires trunk and core strength and stability, neuromuscular coordination, joint range and flexibility and symmetry in movement (Cook et al. 2014). Lower FMS scores are associated with a higher risk of sustaining injuries in specific populations (Kiesel, Plisky & Voight 2007). However, the association between athletes’ FMS score and injury risk, or sports performance is still under investigation, and it is currently not recommended (Bonazza et al. 2017; Dorrel et al. 2015; Hoover et al. 2020; Koehle et al. 2016).

The FMS DS is frequently included in training and assessment regimes because of the global nature of the movement (Kiesel, Plisky & Butler 2011). Performance on the DS test is an indication of athletes’ capacity to improve overall performance on the total FMS score, and therefore the total FMS score may be related to the DS score (Kiesel et al. 2011). The DS is considered one of the more difficult items on the FMS (Kraus, Doyscher & Schüt 2015) as it requires mobility and stability of the ankles, knees and hips with simultaneous stability of the spine and mobility of the shoulder complex. It falls into the ‘complex movement’ factor when factor analysis divides the FMS into two main factors (Kazman et al. 2014, Koehle et al. 2016). For these reasons, the DS was specifically investigated in our study.

Functional assessment of movement can only be valuable if both the reliability and validity of the assessment or screening tool are established (Maclachlan, White & Reid 2015). Both inter- and intra-rater reliability of the FMS and specifically the DS have been found to be high (Frohm et al. 2012; Krosshaug et al. 2007), but several studies have raised concerns about aspects of validity (Bonazza et al. 2017). The items of the FMS have been shown to have poor internal consistency, and the validity of the sum score as a unidimensional construct is questionable (Kazman et al. 2014). It has been suggested that clinicians should rather use the individual item scores of the FMS as a basis for intervention, as each may represent a unique construct (Kazman et al. 2014). However, investigation to establish criterion-related or construct validity of individual items of the FMS is an emerging field. The DS, the hurdle step, the in-line lunge and rotatory stability exercise have all demonstrated fair correlation with components of the Star Excursion Balance Test (Chang et al. 2020), which supports the construct that these items in the FMS may, at least in part, assess neuromuscular contributors to balance. There is a need for more research of this nature. Criterion-related validity of the individual item (e.g., the DS) score also depends on the validity of the rating scale and the accuracy with which it can be used. Observer rating of the FMS DS relies on visual assessment of alignment of the trunk and limbs using specific anatomical landmarks. While the intra-and inter-observer reliability has been established (Frohm et al. 2012; Krosshaug et al. 2007), evidence validating the FMS observer scores against more objective measures such as joint range and kinematic movement analysis is limited with many methodological differences (Butler et al. 2010; Heredia et al. 2021; Hincapié et al. 2022; Scibek, Moran & Edmond et al. 2020; Whiteside et al. 2016).

Butler et al. (2010) and Heredia et al. (2021) found kinematic differences in the hip and knee peak flexion angles and excursion between groups of participants with different scores for the DS. In both these studies, the kinematic analysis was conducted separately from observation and scoring. Scibek et al. (2020) attempted to establish the correlation of DS scores based on kinematic measures and DS scores allocated by video observation and showed poor agreement between kinematic and observer scores. Joint angles measured by manual goniometry show correlation with scores allocated by video analysis (Hincapié et al. 2022). None of these studies conducted real-time scoring of the same movement on which the kinematic measures were based, which may yield different results.

When assessing the DS according to the criteria of the FMS, it is expected that the observer will note deviations in the sagittal and frontal planes using multiple criteria. In the sagittal plane, the observer must note the position of the heels relative to the floor, the position of the torso relative to the tibia or the vertical, the relationship of the femur to the horizontal and the alignment of the knees and an overhead dowel to the feet. In the frontal plane, the only criterion that is used for scoring is the alignment of the knees over the feet. Observation in real-time cannot take place in two planes simultaneously. For our study, the real-time observer rating of the DS in the sagittal plane and 2D kinematic analysis was used.

Despite the concerns around validity of the FMS DS, it is a widely used, simple and cost-effective screening test. Further investigation is warranted to establish criterion-related validity of the FMS DS. Our study aimed to compare the sagittal plane kinematics of three groups of participants who obtained different scores for their FMS DS based on real-time observer ratings. The authors hypothesised that there would be between-group differences in sagittal plane kinematics, indicating that scoring the FMS based on real-time observation displays criterion-based validity.

## Methods

A quantitative, cross-sectional, observational study was conducted at the indoor sports facility of a high school. A sample of convenience from a larger study (Martin, Olivier & Benjamin 2017) was invited to participate. The sample size for the larger study was based on assumptions from the findings of Kiesel et al. (2007) with a power set at 72% – 95%. Two of the 27 injury-free pace bowlers included in the larger study did not attend the screening because of personal and transport problems. Another participant could not complete the FMS evaluation because he experienced anterior knee pain during the testing. Seven data sets could not be included in the analysis because of missing markers. Missing markers is a common problem in the optical motion capture of human-body movement (Piazza et al. 2009). The occlusion of markers can lead to significant problems in tracking accuracy. Therefore, only participants for whom full data sets were available, that is all markers required to calculate the relevant angles were visible at the end range of the DS, were included. Seventeen injury-free, male cricket pace bowlers between the ages of 13 and 18 years were thus included in our study.

### Instrumentation and setting

#### OptiTrack^®^ motion capture system

Multi-camera motion analysis is considered the gold standard in kinematic assessment (Miller & Callister 2009). The OptiTrack^®^ motion capture system has been shown to be as reliable as more expensive systems (Kertis et al. 2010; Montusiewicz et al. 2016). Ten digital high-speed OptiTrack^®^ V100:R2 cameras (NaturalPoint Inc., Oregon, United States [US]), lens type: 10 × 4.5 mm M-Mount, were mounted in an indoor venue. The environment, including time of day, and setting remained unchanged for the duration of the data-collection period. Kinematic data were collected at 100 Hz (at 100 frames per second) using Arena software (Natural Point, Inc., Oregon, US). Identification of marker trajectories, processing and analysis was conducted using Matlab (Version 7.2, The Matworks, Inc., Natick US).

#### Standard Functional Movement Screen equipment

The standard FMS equipment (1.2 m dowel and 5 cm × 15.24 cm board) was used during the performance of the DS (Cook et al. 2014).

### Data-collection procedures

#### Functional Movement Screen and kinematic data capture and processing

**FIGURE 1:**
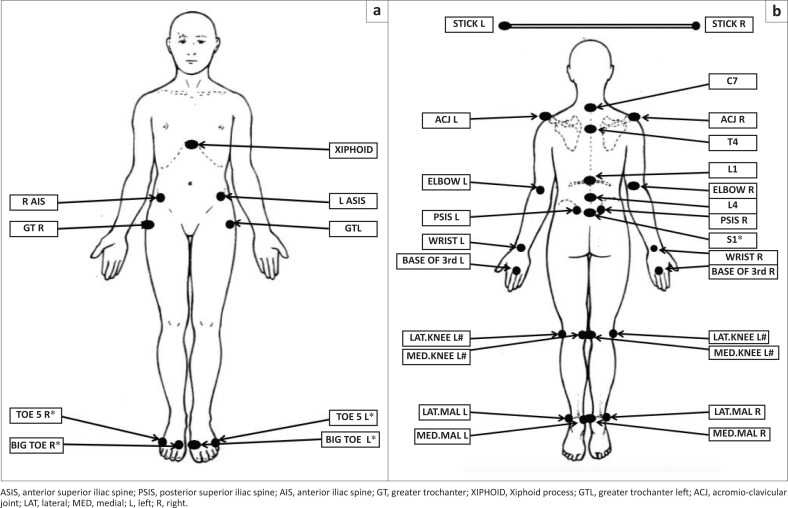
Anatomical landmarks for the placement of light reflective markers.

Motion analysis data were collected to extract kinematics during the performance of the DS movement. Reflective markers were attached with double-sided adhesive tape to pre-determined sites to track movements using a group of cameras. The anatomical landmarks were all bilateral as follows: base of 1st and 5th metatarsals, medial and lateral malleoli of the ankles, medial and lateral condyle of the femur (knee), greater trochanter, anterior superior iliac spine (ASIS) and posterior superior iliac spine (PSIS), acromion, elbow, wrist, knuckle of the third finger and spinous processes of C7, T4, L1, L4 and S1 (Schneiders et al. 2011). Landmarks were selected for their biomechanical and functional significance (Schneiders et al. 2011). The marker placement is demonstrated in Figures 1a and b.

The participants were then asked to perform the DS task of the FMS at a self-selected speed, according to the standard instructions described by the authors of the FMS (Cook et al. 2006). Operational procedures during testing (placement of markers, giving movement instructions and monitoring on-screen tracking of the movements) were conducted by the first author and an assistant, who were experienced in using the FMS and motion capture system. Scoring was conducted by a physiotherapist experienced in the use of the FMS. While the FMS mentions the plane in which movements occur during the DS, it does not describe any specific way in which the observer has to position him or herself when viewing the movement. Therefore, the position in which the observer or physiotherapist placed herself was left up to her discretion. The DS task was scored according to the FMS’s standardised instructions and scoring criteria (Cook et al. 2014). All movements were performed three times, and the observer or physiotherapist allocated the participant to a group based on the highest score. The take with the highest score was also used for kinematic analysis.

If a participant was able to complete the DS as instructed and meet all criteria described in Box 1, a score of 3 was given, and the participant was allocated to Group 3. If the participant was unable to complete the DS as instructed, a 5.00 cm × 15.24 cm board was placed under the heels and the participant was asked to attempt the movement again. If the participant was then able to complete the movement meeting the prescribed criteria, a score of 2 was given, and the participant was allocated to Group 2. If the participant was still unsuccessful, a score of 1 was given, and the participant was assigned to Group 1. In summary, participants were classified into three groups based on the observer score. Those who scored 3, 2 or 1 were allocated to groups labelled Group 3, Group 2 and Group 1, respectively.

BOX 1 Criteria and standardised instructions for overhead deep squat performance on the Functional Movement Screen (Cook et al 2014).

**Table:** 

**Instructions** Stand tall with your feet approximately shoulder width apart and toes pointing forward Grasp the dowel in both hands and place it horizontally on top of your head so your shoulders and elbows are at 90 degrees. Press the dowel so that it is directly above your head. While maintaining an upright torso, keeping your heels flat and the dowel in position, descend as deep as possible. Hold the descended position for a count of one, then return to the starting position.	
**Aspects related to scoring criteria**	**Criteria**
1. Heels remain flat on ground (for score of 3) or board (for score of 2). 2. Upper torso is parallel with tibia or toward vertical. 3. Femur below horizontal (for score of 3 and 2). 4. Knees are aligned over feet in frontal plane. 5. Dowel remains overhead and aligned over feet.	a c b d

During the squat performance, 3D movements of the 36 body landmarks were tracked and captured using the OptiTrack^®^ system. Four measurements related to multiple joint positions in the sagittal plane were calculated on the left (see ‘Aspects related to scoring criteria’ in Box 1). The angles are calculated as the degree of separation between the two segment planes in 2D. All angles and measurements were calculated at the deepest point of descent of the squat.

### Statistical analyses

**FIGURE 2:**
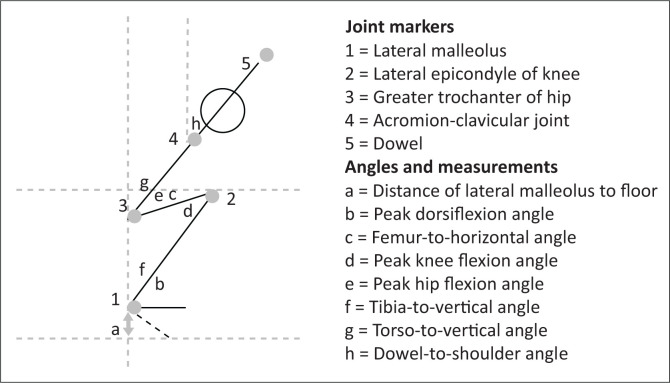
Joint markers and angles used for quantifying scoring criteria.

Specific marker distances, joint angles and angle ratios were used to quantify each scoring criteria (Figure 2). The rationale for measurements used for each of the criteria and the markers used to determine measurements are summarised in Table 1. All calculated ratios (torso-to-vert:tibia-to-vert ratio and dowel-to-shoulder:dowel-to-trochanteric angle ratio) indicate the degree to which two segments are parallel to each other. A 1:1 ratio is indicative of complete parallelism. Means and standard deviations (s.d.) of the kinematic measurements (joint angles and marker distance) were calculated per group.

**TABLE 1:** Rationale for measurements used in the quantification of each of the criteria.

Criteria	Measurements used to quantify criteria	Rationale
1. Heels and feet remain flat on floor or board	*Relative angle height* Distance from floor/board to lateral malleolus marker (cm)/Total standing body height (cm) x 100	As required by the scoring criteria, participants should keep heels flat on the floor or board. As the heels are raised by the board, relative ankle height for those that score 2 and 1 would increase. Relative ankle height for those that score 3 would therefore always be less than those that score 2 or 1.
	*Peak dorsiflexion angle* Formed by the markers on the lateral condyle of the femur (knee), lateral malleolus and base of the 5th metatarsal.	During the performance of a squat where the heel is kept flat on the ground, an increase in hip and knee flexion will result in an increase in dorsiflexion at the ankle. The peak dorsiflexion angle is therefore dependent on hip and knee flexion angle. Considering the scoring criteria, it can be assumed that the knee flexion angle, and therefore also dorsiflexion angle of Group 3 and 2 would exceed that of Group 1. A distinction between Group 3 and 2 would not be so simple. In the starting position the dorsiflexion angle of Group 3 would exceed that of group 2 as the heels of group 2 is raised by the board. At the deepest point of decent this might not be the case. In both groups 3 and 2, the femur passes the horizontal BUT the board might enable a Group 2 participant to decent lower (therefore creating greater knee flexion angle and in turn greater peak dorsiflexion angle) than a Group 3 participant. As the dorsiflexion angle is so dependent on hip and knee flexion angle these measurements were not included in the results.
2. Femur below horizontal	*Femur-to-horizontal angle* Angle formed by the horizontal line and line between the greater trochanter and lateral epicondyle markers which represents the femur.	If the hypothetical horizontal line is set at 90°, participants that score 3 or 2 (i.e., femur below the horizontal) would have a femur-to-horizontal angle of more than 90°, whereas those that score 1 would have a femur-to-horizontal angle of less than 90°.
	*Peak knee flexion angle* Angle formed by line between greater trochanter and lateral epicondyle of the knee, representing the femur, and line between lateral knee epicondyle and the lateral malleolus, representing the tibia.	The peak knee flexion angle for those that score 3 and 2, (where the femur is below the horizontal) would have a greater peak knee flexion angle compared to those that score 1 (where femur is above the horizontal).
	*Peak hip flexion angle* Angle formed by line between greater trochanter and acromion clavicular joint markers, representing the torso and line between greater trochanter and lateral epicondyle markers, representing the femur.	During the performance of a squat where the heel is kept flat on the ground, hip flexion will increase as knee flexion increase (Olson et al. 2011). The peak hip flexion angle of participants that score 3 would therefore have be greater compared to those that score 2 or 1 and those that score 2 greater than those that score 1.
3. Upper torso remains parallel to the tibia or close to vertical	*Torso-to-vertical angle* Angle formed by the torso (represented by line between acromio-clavicular join (ACJ) marker and greater trochanter) and the vertical.	If the hypothetical vertical line is set at 0°, participants that score 3 or 2 would have a smaller torso-to-vertical angle than those that score 1.
	*Tibia-to-vertical: Torso-to-vertical angle ratio* Ratio indicating the degree to which these two lines are parallel to each other. Tibia-to-vertical angle is the angle formed between the vertical and the line between the lateral malleolus of the ankle and lateral epicondyle of the knee, which represents the tibia.	For participants that score 3 and 2 the *tibia-to-vertical: torso-to-vertical angle ratio* should be 1:1 which would indicate that that the torso was kept parallel to the tibia.
4. Dowel should remain overhead	*Dowel-to-shoulder: Dowel-to-trochanteric angle ratio* Ratio indicating the degree to which the dowel-to-shoulder and dowel-to-trochanter lines remain parallel. This would give an indication if the dowel remained overhead at the deepest point of the squat. Dowel-to-shoulder angle is the angle between the vertical and the line between the markers on the dowel and ACJ. Dowel-to-trochanteric angle is the angle between the vertical and line between the markers on the dowel and greater trochanter.	In the starting position the dowel is placed overhead as per instructions given. When viewed from the side the marker on the dowel, ACJ and greater trochanter would be in a straight line or dowel-to-shoulder-angle would be slightly less than the shoulder-to-trochanter angle. If the dowel was kept in the same position when the squat was performed without lumbar flexion (therefore for a score of 3 or 2) the *dowel-to-shoulder: dowel-to-trochanter angle ratio* would be closer to 1:1 than if the dowel was moved forward in which case the dowel-to-shoulder angle would be greater than the dowel-to-trochanter angle.

Demographic and anthropometric data, as well as kinematic measurements of the three groups as well as group pairs, were compared. The Shapiro–Wilks test indicated that data were skewed, and non-parametric tests were therefore used for comparison. The Kruskal–Wallis test, with ties correction, was used for the comparison of data from the three groups. The two-tailed Mann–Whitney U test was used as a post-hoc test for group pair comparisons (*p*-value) of anthropometric and demographic data and kinematic variables between the groups. The effect size (r) was calculated to compare kinematic variables between Groups 1 and 2, Groups 2 and 3 and Groups 1 and 3.

### Ethical considerations

Ethical clearance to conduct our study was obtained from the University of the Witwatersrand Human Research Ethics Committee (Medical) (No. M130657). Written informed consent was obtained from participants who were 18 years of age, while informed assent and consent were obtained from the participants and their parents, respectively, if younger than 18 years.

## Results

Six participants scored three on the DS and were allocated to Group 3. A further six participants scored 2 (Group 2), and five participants scored 1 (Group 1). None of the participants who were allocated to Group 2 or Group 1 were divided based on frontal plane criteria alone (knee medial over the foot). They all displayed sagittal plane criteria that ultimately dictated their score.

### Demographic and anthropometric data

Results related to demographic and anthropometric data are summarised in Table 2. Significant differences in height (*p* = 0.02; *r* = 0.45) and body mass index (BMI) (*p* = 0.03; *r* = 0.31) among the three groups were noted. Post-hoc tests revealed that the height difference was significant between Group 1 and Group 2 (*p* = 0.01; *r* = 0.83), with Group 2 being 11.57 cm taller than Group 1. The small sample size made the BMI difference between Group 2 and Group 1 insignificant, but Group 2 still had a significantly lower BMI (*p* = 0.05; *r* = 0.56) than Group 3.

**TABLE 2:** Demographic and anthropometric comparisons between groups.

Variable	Group 3 (*n* = 6)			Group 2 (*n* = 6)			Group 1 (*n* = 5)			Kruskal- Wallis (*p*-value)	CLES (r) (h2)	Mann-Whitney-U					
												Group 3 vs 2		Group 3 vs 1		Group 2 vs 1	
	Mean ± s.d.	Median	Range	Mean ± s.d.	Median	Range	Mean ± s.d.	Median	Range			*p*-value	effect r	*p*-value	effect r	*p*-value	effect r
Age (years)	16.67 ± 1.03	17	16–18	16.50 ± 0.55	16.5	16-17	16.40 ± 1.14	16	15–18	0.84	−0.12	0.6	0.15	0.64	0.14	0.77	0.08
Height (cm)	183.00 ± 4.69	183	178–189	189.97 ± 4.97	188.5	185-197	178.40 ± 4.93	180	179–183	0.02*	0.45	0.06	0.54	0.27	0.33	0.01*	0.83
Weight (kg)	79.50 ± 8.09	79	73–92	72.67 ± 5.35	72	65-79	82.60 ± 9.40	84	67–92	0.13	0.15	0.15	0.42	0.46	0.22	0.07	0.55
BMI (kg/m^2^)	23.70 ± 1.83	23.52	21.37–26.88	21.84 ± 3.31	20.45	20.36-28.59	25.89 ± 2.03	25.93	23.18–28.40	0.03*	0.31	0.05*	0.56	0.07	0.55	0.07	0.56

s.d., standard deviation; vs, versus.

* , Statistically significant.

### Kinematic measurements

Descriptive statistics related to kinematic measurements in the sagittal plane are summarised in Table 3. Between-group differences and effect sizes are presented in Table 4. No significant differences existed among groups in measurements related to criteria ‘a’ (heels remain flat on ground or board) and ‘d’ (dowel should remain overhead). There were also no significant differences among the groups regarding peak hip flexion and peak knee flexion angles.

**TABLE 3:** Means, standard deviations, medians and ranges of kinematic measurements.

Measurement	Group 3			Group 2			Group 1		
	Mean ± s.d.	Median	Range	Mean ± s.d.	Median	Range	Mean ± s.d.	Median	Range
**Heels remain flat on ground**									
Relative ankle height (%)	5.06 ± 0.42	5.06	4.43–5.49	5.35 ± 1.00	5.04	4.43–7.29	6.28 ± 1.95	5.33	4.67–9.01
**Femur below horizontal**									
Femur-to-horizontal (°)	94.84 ± 17.73	93.28	72.06–116.29	101.92 ± 9.55	103.82	84.22–110.94	120.92 ± 15.62	119.30	102.95–144.79
Peak knee flexion (°)	150.83 ± 22.88	160.17	104.12–160.17	130.40 ± 27.80	126.53	103.44–160.17	125.80 ± 36.87	133.98	82.54–160.17
Peak hip flexion (°)	155.04 ± 10.95	154.77	141.20–169.84	137.33 ± 52.35	158.48	31.27–167.78	143.02 ± 10.72	143.69	127.86–153.60
**Torso remain parallel to tibia or toward vertical**									
Torso-to-vertical (°)	58.95 ± 4.76	59.87	50.62–64.37	58.40 ± 2.88	58.98	53.46–61.33	68.32 ± 5.36	71.40	59.58–71.99
Tibia-to-vertical: Torso-to-vertical angle (ratio)	0.92 ± 0.10	0.90	0.82–1.09	0.82 ± 0.10	0.83	0.70–0.94	1.15 ± 0.38	1.05	0.81–1.67
**Dowel remain overhead and in line with feet**									
Dowel-to-shoulder: Dowel-to-trochanter angle (ratio)	0.92 ± 0.39	0.94	0.52–1.28	0.56 ± 0.1	0.58	0.37–0.68	2.61 ± 1.91	3.79	0.52–4.22

s.d., standard deviation; °, degree.

**TABLE 4:** Kinematically measured Functional Movement Screen Deep Squat quantification data.

Measurement	Kruskal-Wallis (*p*-value)	CLES^®^ (Ƞ^2^)	Mann-Whitney-U					
			Group 3 vs 2		Group 3 vs 1		Group 2 vs 1	
			*p*-value	effect *r*	*p*-value	effect *r*	*p*-value	effect *r*
**Heels remain flat on ground**								
Relative ankle height (%)	0.72	0.1	0.69	0.12	0.47	0.22	0.58	0.17
**Femur below horizontal**								
Femur-to-horizontal (°)	0.05*	0.28	0.63	0.14	0.04*	0.61	0.03*	0.66
Peak knee flexion (°)	0.40	0.01	0.35	0.27	0.14	0.45	0.58	0.16
Peak hip flexion (°)	0.23	0.06	0.75	0.09	0.14	0.44	0.14	0.44
**Torso remain parallel to tibia or toward vertical**								
Torso-to-vertical (°)	0.03*	0.37	0.52	0.18	0.02*	0.66	0.02*	0.72
Tibia-to-vertical: Torso-to-vertical angle (ratio)	0.19	0.09	0.11	0.46	0.11	0.72	0.14	0.44
**Dowel remain overhead and in line with feet**								
Dowel-to-shoulder: Dowel-to-trochanter angle (ratio)	0.40	0.01	0.37	0.20	0.46	0.22	0.28	0.36

s.d., standard deviation; °, degree.

* , statistically significant.

There was a significant femur-to-horizontal angle difference among the three groups (*p* = 0.05; *r* = 0.28). This difference was specifically between Groups 3 and 1 (*p* = 0.04; *r* = 0.61) and Groups 2 and 1 (*p* = 0.03; *r* = 0.66). The mean femur-to-horizontal angle for all groups indicates that the femur was below the horizonal that is greater than 90 degrees.

There was a significant difference in the torso-to-vertical angle among the three groups (*p* = 0.03; *r* = 0.37). The torso-to-vertical angle for Group 1 was significantly greater than for Group 3 (*p* = 0.02; *r* = 0.66) and Group 2 (*p* = 0.02; *r* = 0.72). This indicates that the torso was flexed significantly more forward, away from the vertical, for Group 1 than for Group 3 and Group 2. Although the torso-to-vertical:torso-to-tibia angle ratio (the ratio used to establish the degree to which the torso remains parallel to the tibia) among the three groups was not statistically significant, this ratio was greater for Group 1 (1.15 ± 0.38), further indicating that the torsos were less parallel to the tibia than Group 3 (0.92 ± 0.10) (*r* = 0.72) and Group 2 (0.82 ± 0.01) (*r* = 0.44). The mean torso-to-vertical angle for Group 3 (58.95 ± 4.76°) and Group 2 wase very similar (58.40 ± 2.88°), and the torso-to-vertical:torso-to-tibia angle ratio for Group 3 (0.92 ± 0.10) indicates that torsos of these participants were more parallel to the tibia than those of Group 2 (0.82 ± 0.10). The above results show that: (1) the torsos of participants in Groups 3 and 2 were more towards the vertical than those of Group 1, (2) the torsos of Group 3 were more parallel to the tibia than Group 2 and (3) the torsos of Group 2 were more parallel to the tibia than Group 1. This indicates there are differences in the degree to which the torso remains upright and parallel to the tibia among Group 1, Group 2 and Group 3.

## Discussion

The developers of the FMS suggest that specific mechanics related to the squat differ between the levels of scoring as determined by the FMS (Butler et al. 2010). Our study set out to investigate the criterion validity of the suggested kinematic changes by comparing the kinematic measurements of participants scoring 3 on the FMS DS (as rated by real-time observation) with those of participants scoring 2 or 1 (as rated by real-time observation).

### Demographic and anthropometric data

While the effect of demographic and anthropometric measures was not related to the aim of our study, some noteworthy observations have implications for further research and clinical application. There were significant differences in height and BMI among the three groups. More specifically, Group 2 was significantly taller than Group 1 (*p* = 0.01), and the BMI for Group 3 was higher than Group 2 (*p* = 0.05). These findings are supported by large (*r* = 0.83) and medium (*r* = 0.56) effect sizes. The BMI for Group 3 was significantly higher (23.7 ± 1.83 kg/m^2^) than that of Group 2 (21.84 ± 3.31 kg/m^2^). The mean BMI scores for both groups were still considered within normal ranges (17.2 kg/m^2^ – 24.7 kg/m^2^) for both groups’ mean ages (Mei et al. 2002). Small sample sizes prevent any inferences regarding the interaction of BMI and the FMS DS score. The age of this sample indicates that they were likely at varying stages of pubertal and post pubertal growth. As no measures of musculoskeletal maturity and stage of growth spurt were taken, no inferences can be made regarding the effect of musculoskeletal growth on FMS scores. The growth stage and timing of growth directly affect FMS performance (Portas et al. 2016). In our study, the lowest scoring group (Group 1) had the highest BMI. While overweight and obesity have been associated with poorer performance on the FMS (Duncan, Stanley & Leddington Wright 2013), the BMI for Group 1 was still within the normal range.

Motor skills are affected by age at peak height velocity (Portas et al. 2016) and BMI (McGuine 2006, Duncan et al. 2013). These factors should therefore be considered when studies to confirm the FMS’s validity are conducted. It is also known that increased BMI is associated with increased risk of sports injury in adolescents (McGuine 2006). Further research on the association between FMS scores and athletes’ injury risk must consider BMI.

### Kinematic measurements

A comparison of kinematically measured joint angles indicated that, at the deepest point of squat descent, there were no statistically significant differences regarding the position of the ankle or overhead position of the dowel among the three groups. Small and medium effect sizes supported these findings.

Contrary to our original hypothesis, all three groups’ mean femur-to-horizontal angles passed the horizontal. The suggestion by the authors of the FMS (Cook et al. 2006) that those who score higher would be able to squat deeper while maintaining flat heels, either on the ground or the board and that an observer would be able to accurately rate the quality of the movement based on this specific aspect, is therefore not supported by this finding.

During the performance of the squat, one would expect that when a participant performs a deep squat in such a manner that the femur passes the horizontal, knee and hip flexion would be greater compared to that of a participant where the femur does not pass the horizontal unless that participant uses other compensatory movement strategies. Furthermore, in a closed chain, knee flexion would increase as hip flexion increases (Olson et al. 2011). Even though there were significant differences in femur-to-horizontal angles between Groups 3 and 1 and Groups 2 and 1, there were no significant differences in hip and knee flexion angles. This might indicate that participants might utilise other compensatory movement strategies not addressed explicitly in the FMS scoring criteria to gain depth during the deep squat.

The scoring criteria state that for a score of 3 or 2, the upper torso should be toward the vertical or parallel with the tibia. The torso-to-vertical angle of Group 3 and Group 2 was significantly more vertical when compared to that of Group 1. The finding suggests that, per the scoring criteria, there was no significant difference between Groups 3 and 2 when considering the degree to which the trunk is kept toward the vertical, but that Group 1 flexed the trunk significantly more forward. Considering the scoring criteria, one would expect that the degree to which the torso is kept parallel to the tibia for Groups 3 and 2 would be significantly more (i.e., the torso-to-vertical:tibia-to-vertical ratio closer to 1) than for Group 1. There was no significant difference in the torso-to-vertical:tibia-to-vertical angle ratio among the three groups. The large effect size (ES = 0.72), but insignificant difference, of this ratio between Group 3 and Group 1 but not Groups 2 and 1 (ES = 0.44) should be noted. It seems that the degree to which the trunk moves away from the vertical has a greater effect on the observer’s score than the degree to which the torso is kept parallel to the tibia.

Various authors have explored levels of agreement between human visual and objective 2D or 3D motion analysis when assessing single (Ageberg et al. 2010; Thewlis et al. 2013) and two-leg squats (Thewlis et al. 2013). Even though both studies found clinically acceptable results, in terms of accuracy of observer rating, observers had to only base their rating on a single criterion: whether the knee travelled medial to the second toe in the frontal plane. The FMS requires the observer to score the DS based on joint movement in both the frontal and sagittal planes but does not describe a standard position in which observers should place themselves. Furthermore, the FMS scoring criteria require the observer to simultaneously consider multiple joint movements when rating the quality of the deep squat, which can be challenging for the observer.

All participants were allocated scores based on real-time observation, and all participants displayed sagittal plane criteria that determined their scores. This allowed for statistical analysis comparing 2D kinematic variables among groups based on real-time observer scores. If any participants had been allocated to their group based on a frontal plane criterion alone, then 2D kinematic analysis would have been insufficient. In a clinical setting, observation would occur in the frontal and sagittal plane as the patient performs the movement more than once, and there are multiple criteria to note simultaneously. Future investigations should incorporate participants with frontal plane criteria and comparison with 3D kinematic analysis.

The validity of the FMS DS as a screening test depends on each scoring criterion’s validity. Our findings only support the validity of the standard criteria ‘Upper torso is parallel with tibia or toward vertical’.

### Limitations and implications for further research

Our results should be interpreted with caution as a Type II error may have occurred, and a significant effect could have been missed because of the small sample size (Banerjee et al. 2009; Verrill & Durst 2005). However, effect sizes were also calculated to compensate for a possible type II error.

There is a need for the concurrent validity of the DS to be assessed by correlating an individual’s real-time observations with their 3D kinematic analysis in the sagittal and frontal planes using correlation coefficients. Additionally, the influence of anthropometric measurements and stage of growth in subadult populations on the performance of the FMS warrants further investigation.

## Conclusion

Our study suggests that during the performance of the DS, kinematic differences exist among participants with different scores allocated by real-time observation. Not all the criteria, as described in the FMS and perceived by the observer, are supported by objective kinematic analysis. Our study highlights the potential importance of the torso-to vertical angle when observing the DS from the side. This may assist clinicians to focus and prioritise their observations when conducting the FMS.

A lower score on the FMS DS may be associated with dysfunction, but the pattern of dysfunction cannot be assumed from the scoring criteria. A key criterion in the sagittal plane appears to be ‘torso towards vertical’.
